# Examining the Relationship between Performance on Pre-Clerkship Exam Metrics and Interrupted Academic Progress

**DOI:** 10.1007/s40670-026-02893-3

**Published:** 2026-10-01

**Authors:** Michelle Rogers-Johnson, Thapelo Whitfield, Monica Ethirajan

**Affiliations:** 1 Academic Affairs, Eastern Virginia Medical School at Old Dominion University Norfolk, Norfolk, VA USA; 2 East Carolina University, Greenville, NC USA

**Keywords:** Pre-clerkship exams, Medical knowledge, Assessments, Interrupted academic progress, USMLE Step 1

## Abstract

This retrospective study examines the relationship between performance on pre-clerkship exam metrics and interrupted academic progress (IAP) within the context of a pass/fail grading system and pass/fail Step 1 scoring. For this study, IAP refers to a disruption in the expected transition from the pre-clerkship phase to clinical clerkships, characterized by failure to meet Comprehensive Basic Science Exam (CBSE) and/or Step 1 milestones on schedule or a delay in starting clinical clerkships. Data from cohorts matriculating between 2019 and 2021 were analyzed using exam z-scores, quartile rankings, cumulative averages, cumulative deviation scores, and exam failure counts. Results indicated that students experiencing IAP had lower cumulative z-scores (≤ -1.00), more frequent bottom quartile placements, cumulative exam averages below 81%, three or more exam failures, and/or cumulative deviation scores two standard deviations below cohort means. Logistic regression confirmed these metrics as significant predictors of IAP. These findings suggest that in a pass/fail context, exam metrics remain valuable in identifying students at-risk for IAP.

**Supplementary Information:**

The online version contains supplementary material available at https://doi.org/10.1007/s40670-026-02893-3.

## Introduction

Pre-clerkship medical knowledge exams should play an integral role in assessing student understanding of basic science concepts and supporting preparation for Step 1 and clinical clerkships. Yet, research suggests that these assessments do not consistently predict students’ future performance with accuracy [1]. Puri, McCarthy, and Miller (2022) identified a disconnect between students’ performance in the pre-clerkship curriculum and their performance on Step 1. Performance on most of the pre-clerkship exams had poor correlations with student Step 1 scores, with exams failing to identify students who were at risk for failing the exam. This resulted in missed opportunities for early identification and intervention and increased risk of students having inflated sense of preparation. The researchers found that National Board of Medical Examiners (NBME) Customized Assessments that incorporated higher-order, integrated questions were more predictive of Step 1 outcomes.

This study examines the relationship between performance on pre-clerkship exam metrics and interrupted academic progress (IAP) within the context of a pass/fail grading system and pass/fail Step 1. The authors conducted a retrospective study comparing patterns in exam z-scores, frequency of bottom-quartile placement, cumulative exam averages, cumulative deviation scores, and exam failure counts among students who experienced IAP and their peers. The primary purpose of this study was to identify performance thresholds that could be used to flag students at risk for IAP, defined by the following outcomes of interest: (1) passing a Comprehensive Basic Science Exam (CBSE) or sitting for Step 1 after the Block 1 start date for clinical clerkships, (2) a Step 1 failure, and/or (3) delayed start of clinical clerkships.

## Literature Review

Several studies have examined pre-clerkship exam metrics associated with subsequent academic outcomes [2–8]. For example, Yates (2011) identified that three or more exam failures and pre-clerkship cumulative averages below 50% were significant predictors of IAP. However, the study’s findings may have limited generalizability to schools with differing curricula. Additionally, Yates acknowledged that the study did not account for students who passed exams but exhibited marginal performance, underscoring the need for a more nuanced approach to identifying at-risk students. Building on these insights, Gullo et al. (2015) found that pre-clerkship exams explained a significant proportion of the variance in Step 1 outcomes. Similarly, Krupat et al. (2017) demonstrated that frequent placement in the bottom quartile during first-year exams predicted weaker performance on licensure exams, clerkship grades, and an objective structured clinical exam.

Further advancing this understanding of pre-clerkship exam metrics, Cendán and colleagues (2018) emphasized the importance of longitudinal performance monitoring relative to cohort means. Their findings showed that students who consistently performed significantly below the mean were at a markedly higher risk of IAP. More recently, Wang, Laird-Fick, Parker, Liao, and Solomon (2022) highlighted the predictive power of growth patterns during pre-clerkship years. Their research revealed that students with the lowest scores on the first exam (average = 33.67%) and slow improvement (mean slope = 2.69) faced the greatest risk of failing Step 1. Collectively, this literature suggests the association of various exam metrics with academic difficulty.

However, much of this research was conducted before changes to Step 1 exam cut scores and reporting. In 2022, Step 1 transitioned to pass/fail reporting and the cut-score increased from 194 to 196 [9]. Following the transition to pass/fail Step 1 reporting, there was a notable decline in national first-time pass rates. In 2021, the national first-time pass rate was 96%. By 2024, the first-time pass rate declined to 91% [9, 10]. Although the 2025 first-time pass rate increased to 93%, the decline observed following the transition remains notable. At the same time, the combination of pass/fail grading during the pre-clerkship phase and pass/fail Step 1 reporting may be influencing how students approach learning and assessment. Students may engage less frequently in deep learning strategies and devote less time to exam preparation in favor of activities that distinguish their residency applications [11–15]. Although this shift toward developing well-rounded residency applications aligns with increasingly holistic residency selection processes, it raises questions about whether pre-clerkship exam metrics continue to provide sufficient information about students’ academic progress and preparation for Step 1 and to start clinical clerkships.

Furthermore, limited research has examined which specific exam metrics and performance thresholds are most useful for identifying students at risk for academic difficulty within this contemporary assessment and grading context. Spier et al. (2026) used a survey to examine student-specific factors associated with passing Step 1 by the end of an institution-prescribed dedicated study period and found that pre-dedicated study habits and exam scores were among the strongest predictors of an on-time pass. Steciuch and colleagues (2026) conducted a multi-institutional study comparing Step 1 predictive methods across 12 medical schools in a pass/fail Step 1 environment and found that course exam performance was the second most frequently included predictor across the models. Collectively, these findings support the continued relevance of pre-clerkship exam performance as an indicator of Step 1 readiness. However, the predictive models and performance indicators used across studies vary significantly, leaving questions about which pre-clerkship exam metrics and performance thresholds are most useful for identifying students who may benefit from early intervention.

### Research Gap

Contemporary research has primarily focused on predicting students at-risk for failing Step 1 or Step 2 CK [2, 16, 17]. This study focuses on IAP, which is a broader outcome that captures information about student progression beyond Step 1 failure. This distinction is particularly important for institutions that link Step 1 completion to progression to clinical clerkships, where difficulty completing Step 1 on schedule may delay students’ transition to clinical training. As medical schools increasingly seek to monitor pre-clerkship performance to identify students who may require additional support, more empirical research is needed to determine which exam metrics and performance thresholds provide useful indicators of academic risk. This study contributes to this emerging literature examining the validity of pre-clerkship exams in pass/fail Step 1 and pass/fail pre-clerkship grading context. Based on the literature review and the local context, the authors examined multiple pre-clerkship exam metrics and performance thresholds in relation to IAP, including exam z-scores, bottom-quartile placement, cumulative exam averages, cumulative exam failures, and cumulative deviation scores. By systematically examining these metrics and thresholds, the study provides empirical evidence that can inform how medical schools evaluate their own assessment data and identify performance patterns that may warrant further academic support.

### Conceptual Framework

The conceptual framework for this study emphasizes examining cumulative and relative performance on pre-clerkship exam metrics, recognizing that students may be at risk for academic difficulty even when they do not fail individual exams. The investigation of exam metrics and performance thresholds is informed by educational measurement theory, which emphasizes the importance of validating the proposed interpretations and uses of exam scores [18, 19]. Specifically, this study examines how performance on different exam metrics provides evidence for inferences about student understanding of basic science concepts and how those inferences inform decisions about which students may benefit from additional support and preparation for Step 1 and/or clinical clerkships.

Guided by this framework, the authors addressed the following research questions:

- Do students who experience IAP in the program perform differently on pre-clerkship exam metrics compared to their counterparts?
- What thresholds for pre-clerkship exam performance metrics are useful for identifying students at-risk for IAP?

The authors hypothesized that students who experienced IAP would demonstrate lower pre-clerkship exam performance than their peers as follows:

- Exam z-scores: Students who experienced IAP will have a greater frequency of exam z-scores at or below 1, 1.5, and 2 standard deviations below the cohort mean.
- Bottom-quartile placement: Students who experienced IAP will have more frequent bottom-quartile placement, with three or more bottom-quartile placements expected to be more common among students who experienced IAP based on prior research.
- Cumulative exam failures: Students who experienced IAP will have a greater number of cumulative exam failures, with three or more exam failures expected to be more common among students who experienced IAP based on prior research.
- Cumulative exam average: Students who experienced IAP will have lower cumulative exam averages.
- Cumulative deviation exam score: Students who experienced IAP will have cumulative deviation exam scores that were significantly more negative.

## Methods

### Program Context

This study examines IAP outcomes for the 450 students who matriculated at Eastern Virginia Medical School (EVMS) between 2019 and 2021 (Cohorts 2023–2025). Cohort sizes ranged from 149 to 151 students.

Students completed a two-year pass/fail pre-clerkship curriculum, with exams primarily composed of National Board of Medical Examiners (NBME) Customized Assessment Services (CAS) questions. The program’s pre-clerkship assessment and grading policy established a score of 68% as the passing standard for exams. Due to curricular changes and COVID-19, exams varied by cohort. The 2023 cohort completed two modules concurrently and seven modules sequentially, totaling 23 exams (20 CAS and 3 faculty-created exams). The 2024 and 2025 cohorts completed all nine modules sequentially, with 21 CAS exams.

### IAP Barriers and Outcomes

For this study, IAP refers to a disruption in the expected transition from the pre-clerkship phase to clinical clerkships, characterized by failure to meet Comprehensive Basic Science Exam (CBSE) and/or Step 1 milestones on schedule or a delay in starting clinical clerkships. IAP did not necessarily require a formal leave of absence; students who were permitted to progress through the curriculum on a modified or extended timeline were also considered to have experienced IAP.

IAP was defined as one or more of the following: passing a CBSE or sitting for Step 1 after the clerkship Block 1 start date, a Step 1 failure, and/or delayed start of clinical clerkships. Students meeting one or more criteria were classified as IAP; all others were classified as non-IAP. Complete outcome data were available for all students.

EVMS’s policy required students to pass the CBSE before sitting for Step 1 and complete Step 1 prior to starting clinical clerkships. However, policies impacting academic progression varied across cohorts due to COVID-19 impact on testing center availability, transition to pass/fail Step 1 reporting, and lessons learned from each cohort. Table 1 summarizes cohort-specific policies related to Step 1 and progression to clinical clerkships.

**Table 1:** Cohort-specific policies related to Step 1 and progression to clinical clerkships

Cohort	Step 1 Policy	Options	Impact of Step 1 Failure on Progression in Clerkship Phase
2023	Pass a CBSE prior to sitting for Step 1; allowed to defer Step 1 until August 2022; waived requirement to complete Step 1 prior to starting clinical clerkships	6-week leave of absence (LOA) for first block to pass CBSE LOA for the entire academic year to pass CBSE and Step 1 Withdraw from the program	Removed from the clerkship; on LOA until sit for Step 1 again
2024	Pass a CBSE prior to sitting for Step 1; sit for Step 1 prior to starting clinical clerkships	6-week LOA for first block to pass CBSE and/or sit for Step 1 LOA for the entire academic year to pass CBSE and/or sit for Step 1 Withdraw from the program	Can finish clerkship; once clerkship is complete, placed on LOA until sit for Step 1 again
2025	Pass a CBSE prior to sitting for Step 1; sit for Step 1 prior to starting clinical clerkships	6-week LOA for first block to pass CBSE and/or sit for Step 1 Additional 6-week dedicated preparation course to pass CBSE and/or sit for Step 1 LOA for the entire academic year to pass CBSE and/or sit for Step 1 Withdraw from the program	Can finish clerkship; once clerkship is complete, placed on LOA until sit for Step 1 again

### Analyses

#### Hypothesis#1: Students Who Experienced IAP will have a Greater Frequency of Exam Z-scores at or Below 1, 1.5, and 2 Standard Deviations Below the Mean

To investigate this hypothesis, student z-scores were calculated for each first attempt exam performance, allowing for comparison of student performance relative to their peers on exams. The authors analyzed the average z-scores by IAP status and compared performance across IAP status using the following thresholds: ≤ -1 SD, ≤ -1.5 SD, and ≤ -2 SDs. A chi-square test of independence was used to analyze the distribution of exam z-scores based on IAP status across three Z-score categories: -1.49 to − 1.00 SD below the cohort mean, -1.99 to -1.50 SD below the mean and 2 + SDs below the mean (≤ -2.00). This analysis was conducted for each cohort and the aggregate data set.

#### Hypothesis#2: Students Who Experienced IAP will have more Frequent Bottom-Quartile Placement, with Three or More Bottom-Quartile Placements Expected to be more Common Among Students Who Experienced IAP

To investigate this hypothesis, for each exam, first attempt scores were divided into quartiles to analyze student performance relative to peers and track performance over time, noting if they fell in the bottom 25% of scores. The number of bottom quartile medical knowledge exam scores was calculated. Student performance was then grouped into four bottom quartile frequency categories: zero, once, twice, 3 or more times. This categorization was consistent with Krupat et al.‘s (2017) methodology and findings that 3 + bottom-quartile placements predicted weaker performance on subsequent knowledge-based and clinical assessments. A chi-square test was performed to analyze differences between the IAP and non-IAP groups across the categories. This analysis was conducted for each cohort and the aggregate data set.

#### Hypothesis #3: Students Who Experienced IAP will have a Greater Number of Cumulative Exam Failures, with Three or More Exam Failures Expected to be More Common Among Students Who Experienced IAP

To investigate this hypothesis, the number of medical knowledge exam failures in the pre-clerkship phase was calculated for each student. This metric included the first attempt and retests that were below passing. For pre-clerkship module exams, a fail was defined as < 68% based on the program’s grading and assessment policy. Each student’s number of exam failures was categorized as follows: 0 exam failures, 1 exam failure, 2 exam failures, 3 + exam failures. This categorization was consistent with Yates (2011) finding that 3 or more exam failures was a predictor of interrupted academic program. A chi-square test was performed to analyze differences between the IAP and non-IAP groups across the categories. This analysis was conducted for each cohort and the aggregate data set.

#### Hypothesis#4: Students Who Experienced IAP will have Lower Cumulative Exam Averages

To investigate this hypothesis and identify a meaningful cumulative exam average threshold associated with IAP, first attempt exam scores were averaged for each student. Prior research demonstrated that a cumulative exam average of 50% was predictive of IAP [8]. However, the authors considered this threshold too low for the current context given the program’s exam cut scores and conducted an exploratory analysis to identify a more meaningful threshold for their local context. Cumulative exam averages were compared between students with and without IAP indicators using Welch’s T-test, analyzed by cohort and overall. In addition, the distribution of cumulative exam averages was reviewed for students who experienced IAP vs. those who did not. This metric was beneficial for both looking at an individual student’s performance over the course of the pre-clerkship phase curriculum and comparing performance across cohorts.

#### Hypothesis #5: Students Who Experienced IAP will have Cumulative Deviation Exam Scores that were Significantly More Negative

To investigate this hypothesis, cumulative deviation exam scores were calculated by summing the difference between an individual student’s performance on first attempt exam scores from cohort means. A positive cumulative deviation indicated that the student performed better than the cohort mean overall, while a negative cumulative deviation indicated the opposite. This approach reflects the assessment point accumulation logic used by Cendán et al. (2018) to track students’ cumulative standing relative to the class mean over time. Because it accounts for exam-to-exam difficulty and cohort shifts rather than collapsing performance into a single average, it can flag a student as at-risk even when their raw cumulative average still appears acceptable.

A Welch’s t test was conducted to examine differences in cumulative deviation scores between the IAP and non-IAP groups. Logistic regression models were then fitted separately for the 2023, 2024, and 2025 matriculation cohorts to examine whether cumulative deviation scores predicted IAP and to identify meaningful cumulative deviation thresholds.

## Results

### Hypothesis#1: Students Who Experienced IAP will have a Greater Frequency of Exam Z-scores at or Below 1, 1.5, and 2 Standard Deviations Below the Mean

Analysis of exam z-scores showed that the average z-score between the IAP and non-IAP group varied significantly. Typically, students who experienced IAP scored 1 or more standard deviations below the mean. Table 2 summarizes the average exam z-score by IAP status.

**Table 2:** Average exam Z-Score by IAP status

Cohort	IAP	Non-IAP
All Cohorts	-1.08	0.20
2023	-0.97	0.25
2024	-1.23	0.18
2025	-1.12	0.16

The chi-square analysis indicates a statistically significant association between IAP status and the distribution of z-scores across all cohorts (*p* < .001). Students in the IAP group were more likely to have z-scores in the lower performance categories compared to peers. This relationship was consistent for each cohort. Table 3 summarizes the chi-square results examining the relationship between the three z-score categories and IAP status.

**Table 3:** Chi-square analysis of Z-score distribution across three categories by IAP status

Cohort		Z-Score Category	IAP(Number of Exam Scores)	Non-IAP(Number of Exam Scores)	Chi-Square Statistic	*P*-Value	Effect SizeCramer’s V	Effect Size Interpretation
All Cohorts		-1.49 to -1.00	250	418	134.23	< 0.001	0.298	Small
		-1.99 to -1.50	211	203				
		≤ -2.00	313	114				
2023		-1.49 to -1.00	116	108	39.32	< 0.001	0.275	Small
		-1.99 to -1.50	86	49				
		≤ -2.00	134	28				
2024		-1.49 to -1.00	69	149	40.85	< 0.001	0.288	Small
		-1.99 to -1.50	67	72				
		≤ -2.00	89	45				
2025		-1.49 to -1.00	65	161	54.18	< 0.001	0.330	Medium
		-1.99 to -1.50	58	82				
		≤ -2.00	90	41				

Although the IAP group comprised fewer students overall, they had more exam scores represented in the lowest z-score range (≤ -2.00). Conversely, students who did not experience IAP were more likely to have exam scores in the higher z-score category (-1.49 to -1.00).

### Hypothesis#2: Students Who Experienced IAP will have more Frequent Bottom-Quartile Placement, with Three or More Bottom-Quartile Placements Expected to be More Common Among Students Who Experienced IAP

A chi-square test showed that students in the IAP group were significantly more likely to score in the bottom quartile three or more times compared to the non-IAP group. This finding was consistent across all three cohorts. Table 4 summarizes the results of the chi-square analysis examining the relationship between frequency in the bottom quartile and IAP status.

**Table 4:** Chi-square analysis of bottom quartile frequency distribution by IAP status

Cohort	Frequency in Bottom Quartile	IAP ( Number of Students)	Non-IAP ( Number of Students)	Chi-Square Statistic	*P*-Value	Effect Size Cramer’s V	Effect Size Interpretation
All Cohorts	0		90	59.01	< 0.001	0.362	Medium
	1		48				
	2		51				
	3+	69	192				
2023	0		28	29.43	< 0.001	0.442	Medium
	1		14				
	2		23				
	3+	31	55				
2024	0		26	15.52	0.001	0.323	Medium
	1		20				
	2		16				
	3+	19	68				
2025	0		36	15.33	0.002	0.320	Medium
	1		14				
	2		12				
	3+	19	69				

While all students in the IAP group scored in the bottom quartile on 3 or more exams, a significant number of students in the non-IAP students also met this threshold. Variability in bottom-quartile cut-scores, influenced by exam difficulty, ranged from 74% to 89.58%, with most falling between 80% and 84%. Narrow score distributions on easier exams may explain some bottom-quartile placements for non-IAP students.

Because a significant percentage of students who had not experienced IAP had 3 or more exam scores in the bottom quartile, the authors reviewed the distribution of bottom quartile frequencies for both groups. The authors observed notable differences in the average frequency of bottom-quartile exam scores between the IAP and non-IAP groups. Across cohorts, students who experienced IAP averaged 15 or more bottom-quartile exam scores, whereas students without IAP averaged 4 bottom-quartile exam scores.

### Hypothesis #3: Students Who Experienced IAP will have a Greater Number of Cumulative Exam Failures, with Three or More Exam Failures Expected to be More Common Among Students Who Experienced IAP

A chi-square test showed that students with multiple exam failures in the pre-clerkship phase were more likely to experience IAP, with students having 3 + failures at the highest risk. X-squared results: *χ²*= 220.62, df = 3, p-value < 0.001). Table 5 summarizes the results.

**Table 5:** Chi square results: Number of exam failures by IAP status

Number of exam failures	IAP *(*Number of Students*)*	Non-IAP *(*Number of Students*)*	Chi-Square Statistic	*P*-Value	Effect Size Cramer’s V	Effect Size Interpretation	AUC (95% CI)
0	14	343	228.98	< 0.001	0.713	Large	0.87 (0.82-0.93)
1	10	27					
2	15	8					
3+	30	3					

The authors conducted a logistic regression analysis to examine whether exam failures predicted IAP status. The overall model was statistically significant (*p* < .001), with a substantial reduction in deviance relative to the null model (ΔDeviance = 174.47, df = 3). Model performance was assessed using the area under the receiver operating characteristic curve (AUC). The exam failures model demonstrated strong discrimination (AUC = 0.87, 95% CI [0.82, 0.93]). Exam failures were strongly associated with increased odds of IAP. Compared to students with no exam failures, students with one exam failure had significantly higher log-odds of IAP status (β = 2.21, SE = 0.46, *p* < .001), students with two exam failures had higher log-odds (β = 3.83, SE = 0.52, *p* < .001), and students with three or more exam failures had higher log-odds (β = 5.50, SE = 0.66, *p* < .001). The model’s AIC (238.1) indicates a relatively good fit.

### Hypothesis#4: Students Who Experienced IAP will have Lower Cumulative Exam Averages

Significant differences were found, with students in the IAP group scoring at least 2 standard deviations below peers across all cohorts and overall (effect sizes > 2.00). This consistent pattern across different years highlights the strong association between lower cumulative exam averages and the likelihood of having an IAP indicator. Table 6 summarizes the differences in cumulative exam averages by IAP status.

**Table 6:** Cumulative exam average by IAP status

Cohort	IAP: Cumulative Exam Average	Non-IAP: Cumulative Exam Average	Welch’s T-Statistic	*P*-value	Effect Size (Hedge’s g)	Effect Size Interpretation
All Cohorts	77.97 (4.72)	88.11 (4.36)	16.61	< 0.001	2.29	Large
2023	77.66 (5.19)	87.54 (4.13)	9.83	< 0.001	2.25	Large
2024	77.43 (3.09)	88.17 (4.57)	13.19	< 0.001	2.42	Large
2025	79.03 (5.29)	88.57 (4.33)	7.51	< 0.001	2.13	Large

**Fig. 1:**
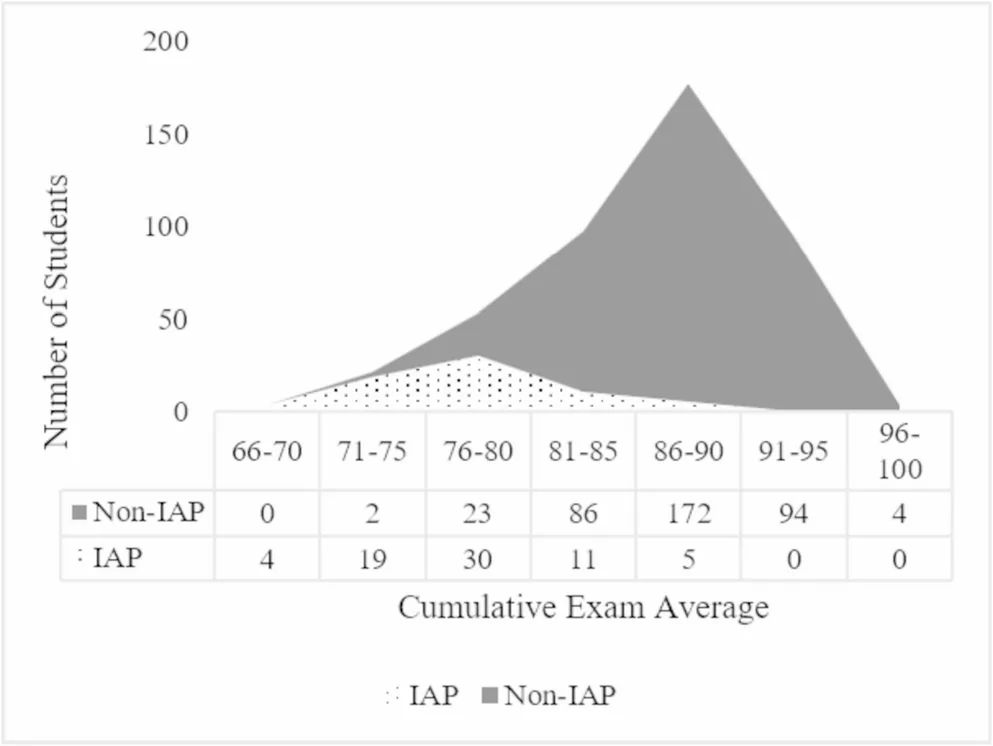
Cumulative exam average distribution by IAP status

The distribution of cumulative exam averages was reviewed for students who experienced IAP vs. those who did not. The graph shows that students who experienced IAP tended to have cumulative exam averages below 81%. In contrast, students who did not experience IAP were heavily concentrated in the higher score ranges, particularly in the 86–90 category. Notably, no student in the IAP group had a score in the two highest score ranges (91–95) and (96–100); no student in the non-IAP group had a score in the lowest score range (66–70). Figure 1 shows the cumulative exam average distribution by IAP status.

### Hypothesis #5: Students Who Experienced IAP will have Cumulative Deviation Exam Scores that Were Significantly More Negative

A Welch’s T-test showed a significant difference in cumulative deviation exam scores between students with interrupted academic progress (IAP) and those without with students in the IAP group scoring at least 2 standard deviations below peers across all cohorts and overall (effect sizes > 2.00). This consistent pattern across different years highlights the strong association between lower cumulative deviation exam scores and the likelihood of having an IAP indicator. Table 7 summarizes these results.

**Table 7:** Average cumulative deviation exam score by IAP status

Cohort	IAP Average Cumulative Deviation Score (SD)	Non-IAP Average Cumulative Deviation Score (SD)	Welch’s T-statistic	*P*-value	Effect Size (Hedge’s g)	Effect Size Interpretation
All Cohorts	-183.55 (103.60)	33.26 (94.14)	16.21	< 0.001	2.26	Large
2023	-180.66 (119.26)	46.67 (94.95)	9.84	< 0.001	2.25	Large
2024	-196.88 (64.99)	28.77 (95.92)	13.18	< 0.001	2.42	Large
2025	-174.94 (111.08)	25.44 (90.97)	7.51	< 0.001	2.13	Large

The cumulative deviation score models showed strong discrimination across cohorts (2023: AUC = 0.93, 95% CI [0.89, 0.98]; 2024: AUC = 0.96, 95% CI [0.93, 0.99]; 2025: AUC = 0.91, 95% CI [0.85, 0.98]). These values indicate that the identified metrics translate into strong classification performance in distinguishing students who experienced IAP from those who did not. After performing the regression, probabilities were plotted using ggplot in R, visually identifying the score at which the probability of having an IAP indicator reached 0.50. Table 8 provides a summary of these results.

**Table 8:** Cumulative deviation exam scores associated with 50% probability of IAP

Cohort	Intercept Coef.	Cum. Dev. Exam Score Coef.	Cum. Dev. Exam Score at 50% IAP Probability	*P*-value	AUC (95% CI)
2023	-2.48***	− 0.02***	- -127.30	< 0.001	0.93 (0.89-0.98)
2024	-4.18***	− 0.02***	-174.55	< 0.001	0.96 (0.93-0.99)
2025	-3.37***	− 0.02***	-167.64	< 0.001	0.91 (0.85-0.98)

**Fig. 2:**
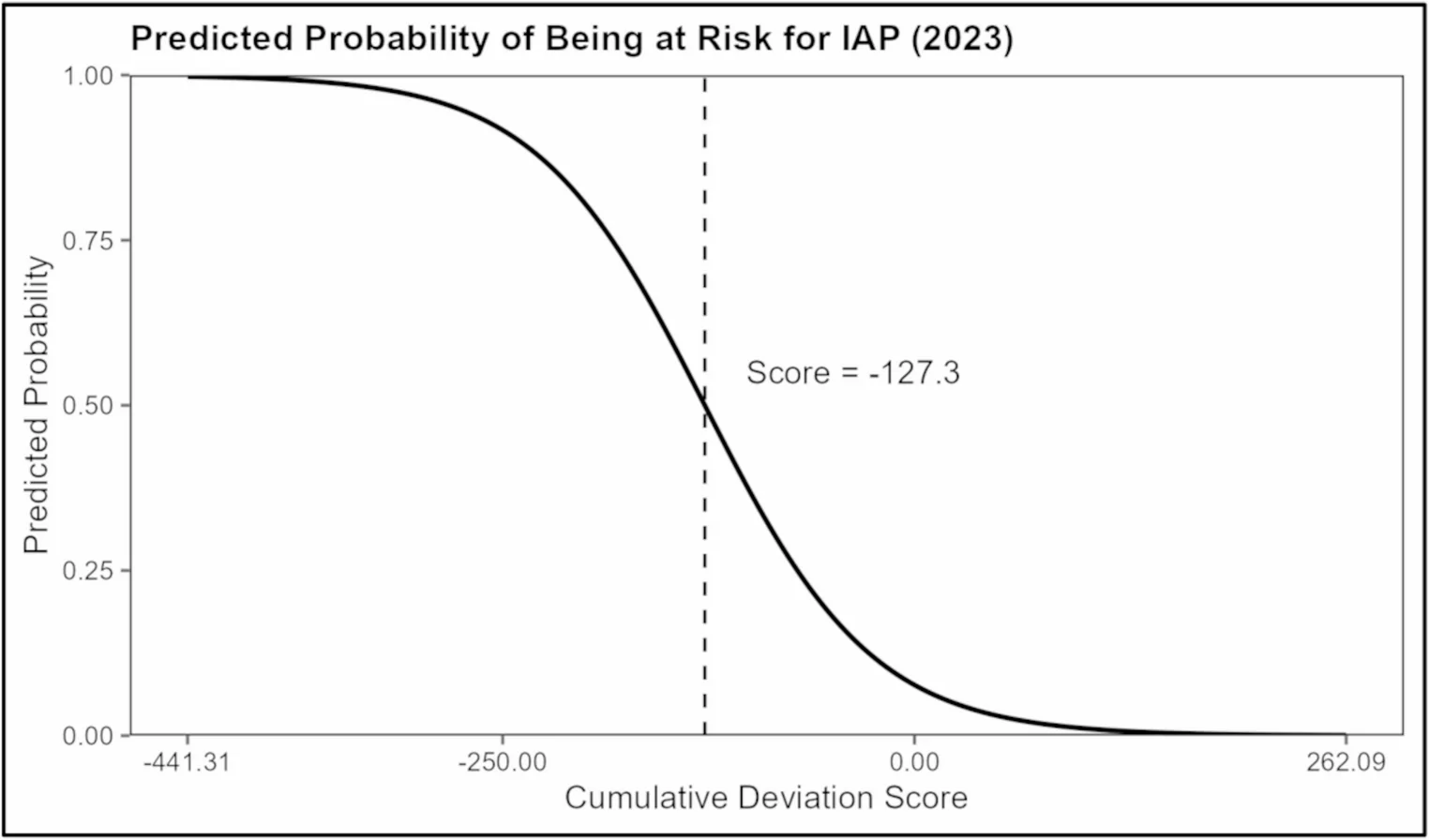
Predicted probability of being at risk for IAP (2023)

**Fig. 3:**
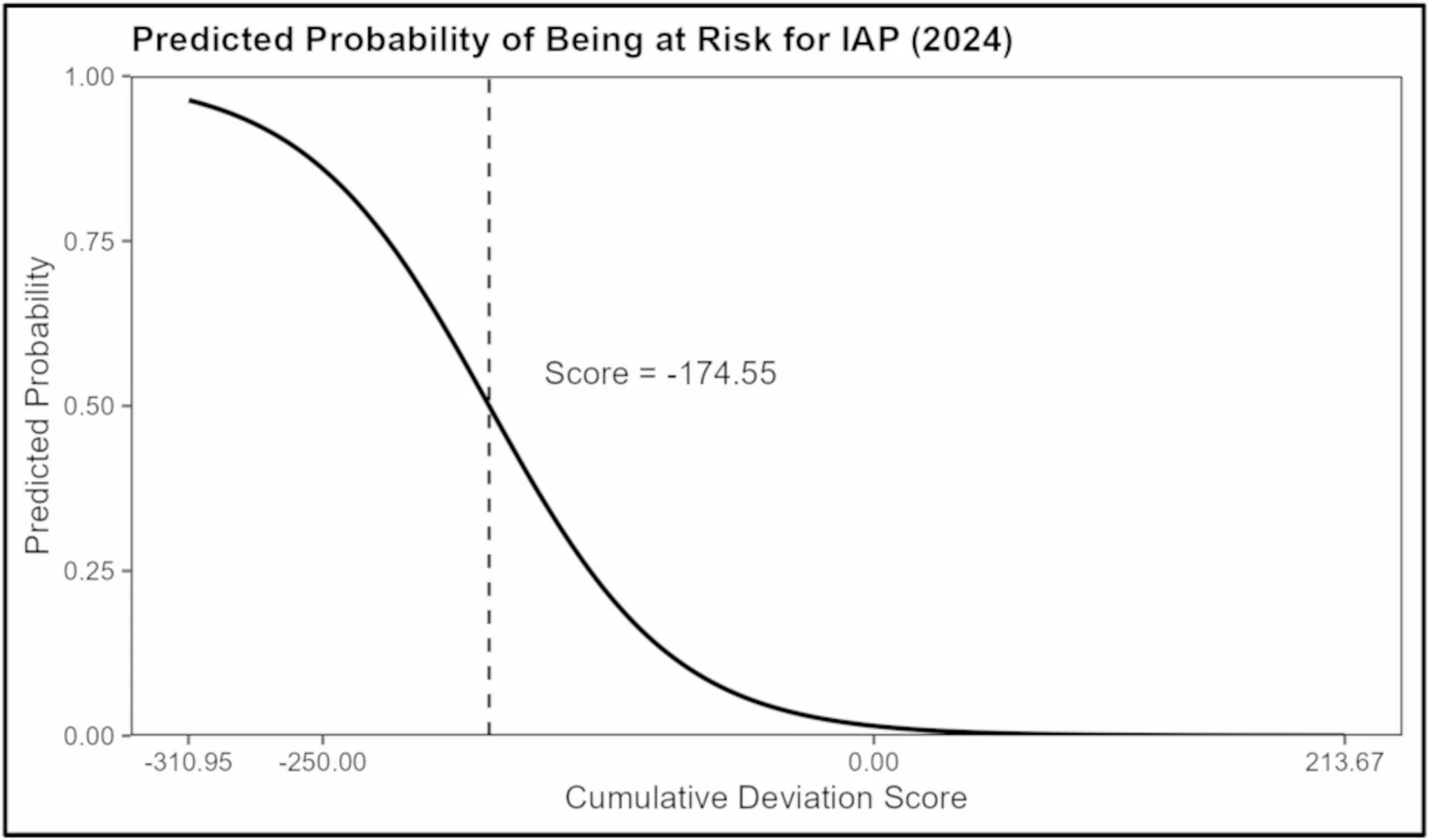
Predicted probability of being at risk for IAP (2024)

**Fig. 4:**
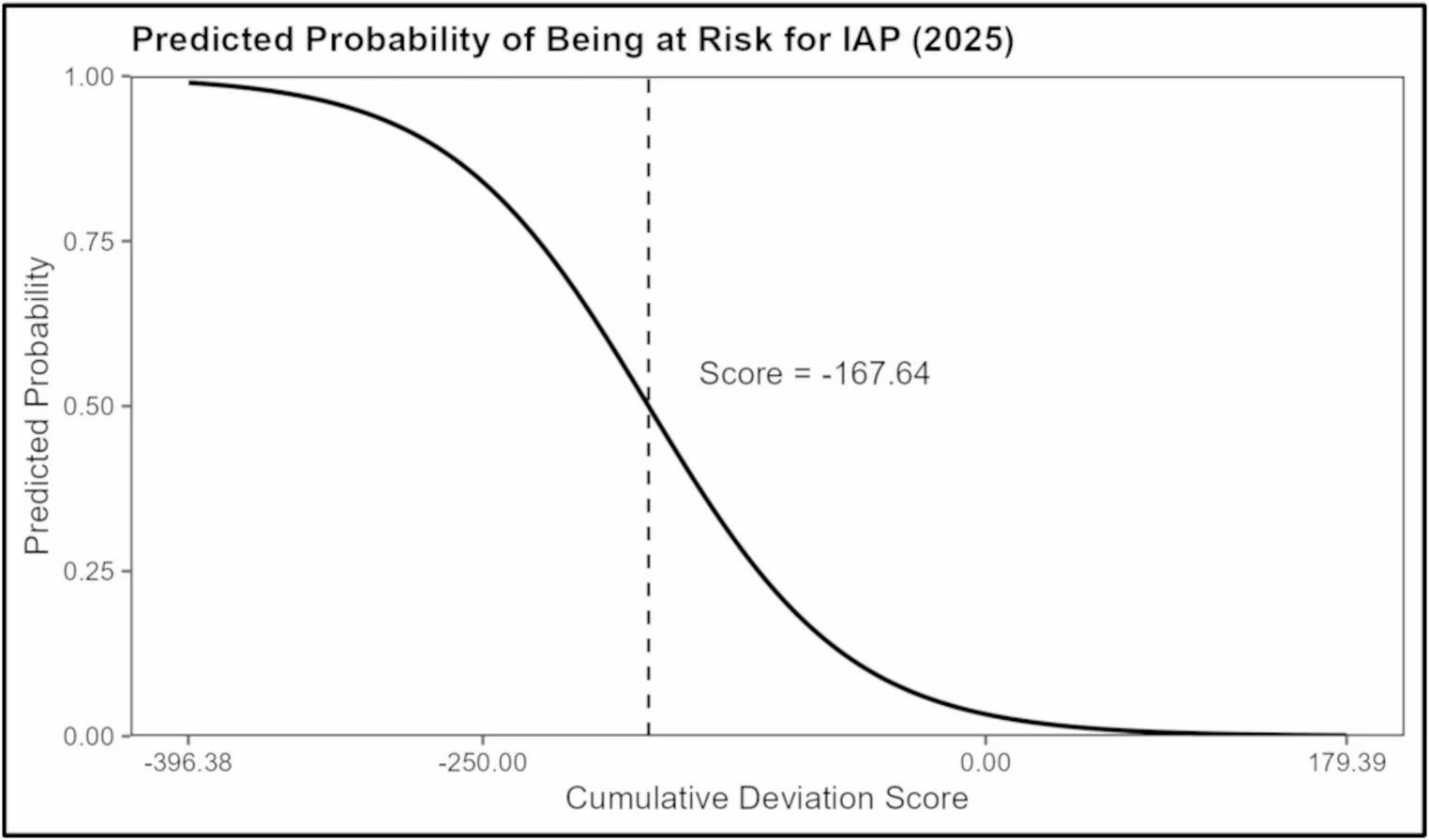
Predicted probability of being at risk for IAP (2025)

For the 2023 cohort, the logistic regression model indicates that each unit decrease in the cumulative deviation score increases the log odds of having an IAP indicator by approximately 2%. The coefficients for the intercept (-2.48) and cumulative deviation exam score (-0.02) were both significant with p-values < 0.001. The cumulative deviation exam score at which the probability of having an IAP indicator reached 0.50 was − 127.30. Figs. 2, 3 and 4 show the predicted probability of being at risk for IAP for each of the three cohorts based on their respective exam cumulative deviation scores.

For the 2024 cohort, the logistic regression model indicates that each unit decrease in the cumulative deviation score increases the log odds of having an IAP indicator by approximately 2%. The coefficients for the intercept (-4.18) and cumulative deviation exam score (-0.02) were both significant with p-values < 0.001. The cumulative deviation exam score at which the probability of having an IAP indicator reached 0.50 was − 174.55.

For the 2025 cohort, the logistic regression model indicates that each unit decrease in the cumulative deviation score increases the log odds of having an IAP indicator by approximately 2%. The coefficients for the intercept (-3.37) and cumulative deviation exam score (-0.02) were both significant with p-values < 0.001. The cumulative deviation exam score at which the probability of having an IAP indicator reached 0.50 was − 167.64.

The cumulative deviation exam score thresholds at which the probability of having an IAP indicator reaches 0.50 was different for each matriculation year. This illustrates that what constitutes a “significant” negative cumulative deviation score for determining IAP risk varies by cohort, influenced by each cohort’s overall performance. For instance, the class of 2023 had a lower cumulative exam average compared to the classes of 2024 and 2025, thereby requiring a less extreme negative deviation to reach the 50% probability threshold for IAP. Specifically, the class of 2023 reached this threshold at a cumulative deviation score of -127.3, while the classes of 2024 and 2025 required more substantial negative deviations (-174.55 and − 167.64, respectively) to reach the same probability of IAP.

This suggests that for cohorts already performing at lower levels, a relatively smaller negative deviation from the norm is sufficient to consider a student at risk for IAP. In contrast, cohorts with higher overall performance require a more pronounced deviation to trigger the same level of concern. As such, determining IAP risk must consider cohort-specific performance levels, as relying on a standardized deviation threshold across all cohorts may overlook students at risk within lower-performing cohorts or overestimate risk in higher-performing ones.

## Discussion

This study examined the relationship between pre-clerkship exam performance metrics and IAP status within a pass/fail pre-clerkship curriculum and a pass/fail Step 1 environment. Across all three cohorts, students who experienced IAP demonstrated lower performance on pre-clerkship exam metrics, including lower exam z-scores, bottom-quartile performance 3 + times, lower cumulative exam averages, more cumulative exam failures, and more negative cumulative deviation scores. Notably, the threshold of three or more bottom-quartile placements did not adequately distinguish between the IAP and non-IAP groups. Although 100% of students who experienced IAP met this threshold, 50% of non-IAP students did as well. In contrast, students who experienced IAP had an average of 15 bottom-quartile exam scores compared with 4 among non-IAP students, suggesting that a higher threshold may provide greater discriminatory value. If institutions use quartile-based metrics to identify students for academic support, higher thresholds may reduce false-positive identification. This distinction is important because false-positive identification may not only increase demands on academic support resources but also raise concerns about the validity and intended use of the metric if it cannot adequately distinguish students who require more intensive intervention from those who may benefit from less intensive support. Collectively, these findings suggest that pre-clerkship exam metrics may provide useful indicators of academic risk during the transition from the pre-clerkship phase to clinical clerkships, but their interpretation and use should be guided by empirically supported thresholds and the intended purpose of the assessment.

The findings also provide insight into potential performance thresholds for identifying students at risk for IAP. Students who experienced IAP were more likely to have exam z-scores at or below the examined thresholds of 1, 1.5, and 2 standard deviations below the cohort mean, three or more bottom-quartile placements, and three or more exam failures. They were more likely to have cumulative exam averages below 81%; they also demonstrated more negative cumulative deviation scores, although the thresholds associated with IAP varied across cohorts. These findings illustrate the value of interpreting assessment performance from multiple perspectives: criterion-referenced metrics identify students who fall below established performance standards, norm-referenced metrics identify students whose performance is relatively low compared with peers, and cumulative measures capture patterns of performance across time. Thus, no single metric should be viewed as a definitive indicator of risk; rather, these measures may provide complementary information to support longitudinal academic advising. The choice and interpretation of thresholds should also consider the intended use of the information, the consequences of intervention, and the feasibility of tracking the metric within institutional data systems. Programs early in tracking stages may prioritize simple metrics (e.g., exam failures, cumulative averages) to efficiently identify at-risk students.

Furthermore, this study highlights the significant role that policies play in supporting student readiness for Step 1 and clinical clerkships. Programs must establish empirically based pass/fail standards and retest policies that prepare students to successfully sit for Step 1 and progress to clinical clerkships. Standards that are too low, risk allowing students to progress through the pre-clerkship curriculum while remaining unprepared for Step 1 and the next curricular phase in their education. Conversely, overly stringent standards can lead to unnecessary failures, negatively impacting students’ transcripts and placing an undue burden on institutional resources due to increased remediation and advising demands. Once exam pass/fail standards are established, they should remain consistent across courses long enough to generate meaningful validity evidence. Similarly, retest policies must balance student support and academic rigor. Overly lenient policies may lead students to underprepare for exams, resulting in delayed Step 1 readiness. Overly stringent retest policies may unnecessarily hinder progress, increasing failure rates and institutional strain.

In summary, effectively using pre-clerkship assessment data to support student readiness for Step 1 and clinical clerkships requires identifying appropriate exam metrics, establishing meaningful performance thresholds, and developing data-informed policies to support student progression. The validity of these metrics depends not only on their relationship with meaningful academic outcomes, but also on the appropriateness of their interpretation and intended use. Continued evaluation of how exam metrics and performance thresholds are interpreted and used can help institutions identify students who may benefit from additional support and ensure that assessment information informs appropriate academic interventions.

## Conclusion

The authors posed two research questions: Do students who experience IAP perform differently on pre-clerkship exam metrics compared with their peers, and what thresholds for pre-clerkship exam performance metrics are useful for identifying students at risk for IAP? The findings demonstrated that students who experienced IAP performed differently on pre-clerkship exam z-scores, exam failures, cumulative exam averages, and cumulative deviation exam scores. Students who experienced IAP were more likely to perform two standard deviations below the mean on individual exams and overall. Their cumulative exam means were typically < 81% average. They were also more likely to have three or more failures. Bottom-quartile placement was also associated with IAP. However, the threshold of three or more bottom-quartile placements did not sufficiently distinguish IAP from non-IAP students. The findings therefore suggest that a higher threshold may be more useful when using bottom-quartile performance to identify students at risk for IAP.

The exam metrics explored in this study provided complementary information about students' relative, criterion-referenced, and longitudinal performance on medical knowledge exams in the pre-clerkship phase. These metrics can assist institutions with identifying students who may benefit from additional program supports before difficulties disrupt progression to clinical clerkships . However, the validity of these metrics will depend on the context in which scores are interpreted and used. Institutions should evaluate exam metrics and performance thresholds against meaningful academic outcomes and with consideration of student-specific factors. Replication across institutions and cohorts is needed to determine the generalizability of these findings, as differences in curriculum design, grading systems, and assessment strategies may influence the utility of these metrics for identifying students at risk for IAP. Overall, these findings demonstrate the potential value performance data on pre-clerkship exam metrics can provide to support student success.

## Supplementary Information

Supplementary Material 1.

## Data Availability

The data used in this study will not be made publicly available. However, inquiries related to the data may be directed to the first author.
