# Supplementary material for: Examining the Relationship between Performance on Pre-Clerkship Exam Metrics and Interrupted Academic Progress
Source: Med Sci Educ. 2026 Oct 1;36(6):2911–21. doi: 10.1007/s40670-026-02893-3 (PMC13759945; doi:10.1007/s40670-026-02893-3)
Supplement: Supplementary file 1 [file 40670_2026_2893_MOESM1_ESM.docx]

Supplemental Figures: Frequency in Bottom Quartile by IAP Status

| **Frequency in Bottom Quartile (All Cohorts)** | **IAP**  **(Number of Students)** | **Non-IAP**  **(Number of Students)** |
| --- | --- | --- |
| 0 |  | 90 |
| 1 |  | 48 |
| 2 |  | 51 |
| 3 | 2 | 24 |
| 4 | 2 | 21 |
| 5 | 1 | 29 |
| 6 |  | 10 |
| 7 | 1 | 16 |
| 8 |  | 22 |
| 9 | 2 | 11 |
| 10 | 2 | 6 |
| 11 | 4 | 7 |
| 12 | 1 | 12 |
| 13 | 5 | 12 |
| 14 | 5 | 4 |
| 15 | 7 | 6 |
| 16 | 4 | 3 |
| 17 | 5 | 4 |
| 18 | 6 | 2 |
| 19 | 6 | 2 |
| 20 | 8 |  |
| 21 | 3 | 1 |
| 22 | 4 |  |
| 23 | 1 |  |

| **Frequency in Bottom Quartile 2023** | **IAP**  **(Number of Students)** | **Non-IAP**  **(Number of Students)** |
| --- | --- | --- |
| 0 |  | 28 |
| 1 |  | 14 |
| 2 |  | 23 |
| 3 | 1 | 5 |
| 4 | 1 | 7 |
| 5 | 1 | 7 |
| 6 |  | 5 |
| 7 |  | 4 |
| 8 |  | 5 |
| 9 | 1 | 6 |
| 10 | 1 | 2 |
| 11 | 3 | 2 |
| 12 |  | 3 |
| 13 | 3 | 4 |
| 14 | 1 | 2 |
| 15 | 3 | 2 |
| 16 | 1 |  |
| 17 | 3 |  |
| 18 | 2 |  |
| 20 | 4 |  |
| 21 | 1 | 1 |
| 22 | 4 |  |
| 23 | 1 |  |

| **Frequency in Bottom Quartile 2024** | **IAP**  **(Number of Students)** | **Non-IAP**  **(Number of Students)** |
| --- | --- | --- |
| 0 |  | 26 |
| 1 |  | 20 |
| 2 |  | 16 |
| 3 |  | 12 |
| 4 |  | 8 |
| 5 |  | 12 |
| 6 |  | 3 |
| 7 |  | 7 |
| 8 |  | 3 |
| 9 | 1 | 1 |
| 10 |  | 1 |
| 11 |  | 2 |
| 12 |  | 5 |
| 13 |  | 5 |
| 14 | 4 | 1 |
| 15 | 3 | 1 |
| 16 | 1 | 2 |
| 17 | 2 | 2 |
| 18 | 2 | 1 |
| 19 | 4 | 2 |
| 20 | 2 |  |

| **Frequency in Bottom Quartile 2025** | **IAP**  **(Number of Students)** | **Non-IAP**  **(Number of Students)** |
| --- | --- | --- |
| 0 |  | 36 |
| 1 |  | 14 |
| 2 |  | 12 |
| 3 | 1 | 7 |
| 4 | 1 | 6 |
| 5 |  | 10 |
| 6 |  | 2 |
| 7 | 1 | 5 |
| 8 |  | 14 |
| 9 |  | 4 |
| 10 | 1 | 3 |
| 11 | 1 | 3 |
| 12 | 1 | 4 |
| 13 | 2 | 3 |
| 14 |  | 1 |
| 15 | 1 | 3 |
| 16 | 2 | 1 |
| 17 |  | 2 |
| 18 | 2 | 1 |
| 19 | 2 |  |
| 20 | 2 |  |
| 21 | 2 |  |
